# Validation and quality assessment of macromolecular structures using complex network analysis

**DOI:** 10.1038/s41598-019-38658-9

**Published:** 2019-02-08

**Authors:** Jure Pražnikar, Miloš Tomić, Dušan Turk

**Affiliations:** 1 Faculty of Mathematics, Natural Sciences and Information Technologies, University of Primorska, Glagoljaška 8, Koper, Slovenia; 2 Department of Biochemistry, Molecular and Structural Biology, Institute Jožef Stefan, Jamova 39, Ljubljana, Slovenia; 3 Center of excellence for Integrated Approaches in Chemistry and Biology of Proteins, Jamova 39, Ljubljana, Slovenia

## Abstract

Validation of three-dimensional structures is at the core of structural determination methods. The local validation criteria, such as deviations from ideal bond length and bonding angles, Ramachandran plot outliers and clashing contacts, are a standard part of structure analysis before structure deposition, whereas the global and regional packing may not yet have been addressed. In the last two decades, three-dimensional models of macromolecules such as proteins have been successfully described by a network of nodes and edges. Amino acid residues as nodes and close contact between the residues as edges have been used to explore basic network properties, to study protein folding and stability and to predict catalytic sites. Using complex network analysis, we introduced common network parameters to distinguish between correct and incorrect three-dimensional protein structures. The analysis showed that correct structures have a higher average node degree, higher graph energy, and lower shortest path length than their incorrect counterparts. Thus, correct protein models are more densely intra-connected, and in turn, the transfer of information between nodes/amino acids is more efficient. Moreover, protein graph spectra were used to investigate model bias in protein structure.

## Introduction

Insight into the three-dimensional structures of macromolecules resolved to atomic detail is crucial for our understanding of biological processes. As only the correct structures can be used in earnest to address biological questions, the validation and quality assessment of three-dimensional structures is an important issue in structural biology^1–4^. Therefore, tools for validating many criteria have been developed, including Ramachandran plot outliers, all-atom clash scores, deviations from bonding geometry, and rotamers. These criteria are good indicators of the local structure’s correctness. Implementation of Huber’s rule that the structure is correct when it fits the electron density and is correct locally and globally relies on human assessment^5^; however, algorithms and their software implementations are lacking.

The informational abstraction of three-dimensional macromolecular structures into residue networks provides a means to address this issue. The analysis and exploration of a complex networks approach are still expanding across various research areas^6^. In the last decade, protein structures have been modelled as networks numerous times^7–9^. Interacting amino acids, presented as nodes and edges, have small-world properties^10^ with relatively short characteristic path lengths and high clustering coefficients. From this perspective, the interacting amino acids are like other self-organized networks, such as World Wide Web pages^11^, biological signaling pathways^12^, metabolic networks^13^, and scientific collaboration networks^14^. Protein and residue network models have been successfully used for predictions of catalytic sites^15–17^, examinations of protein structures from modeling^18^, investigations of protein dynamics^19^, analysis of protein-protein interaction networks^20,21^, graph theoretical analysis of protein pathways^22^, and assignments of residues that play crucial roles in protein folding^23–25^. Surprisingly, to date, residue network characteristics have not been used to validate three-dimensional protein structures deposited in Protein Data Bank and, Protein Graph Repository. Here, we demonstrate the use of complex network analysis for macromolecule model quality assessment and for differentiation between incorrect and correct protein structures.

## Results

### Node degree of residue network models

**Figure 1:**
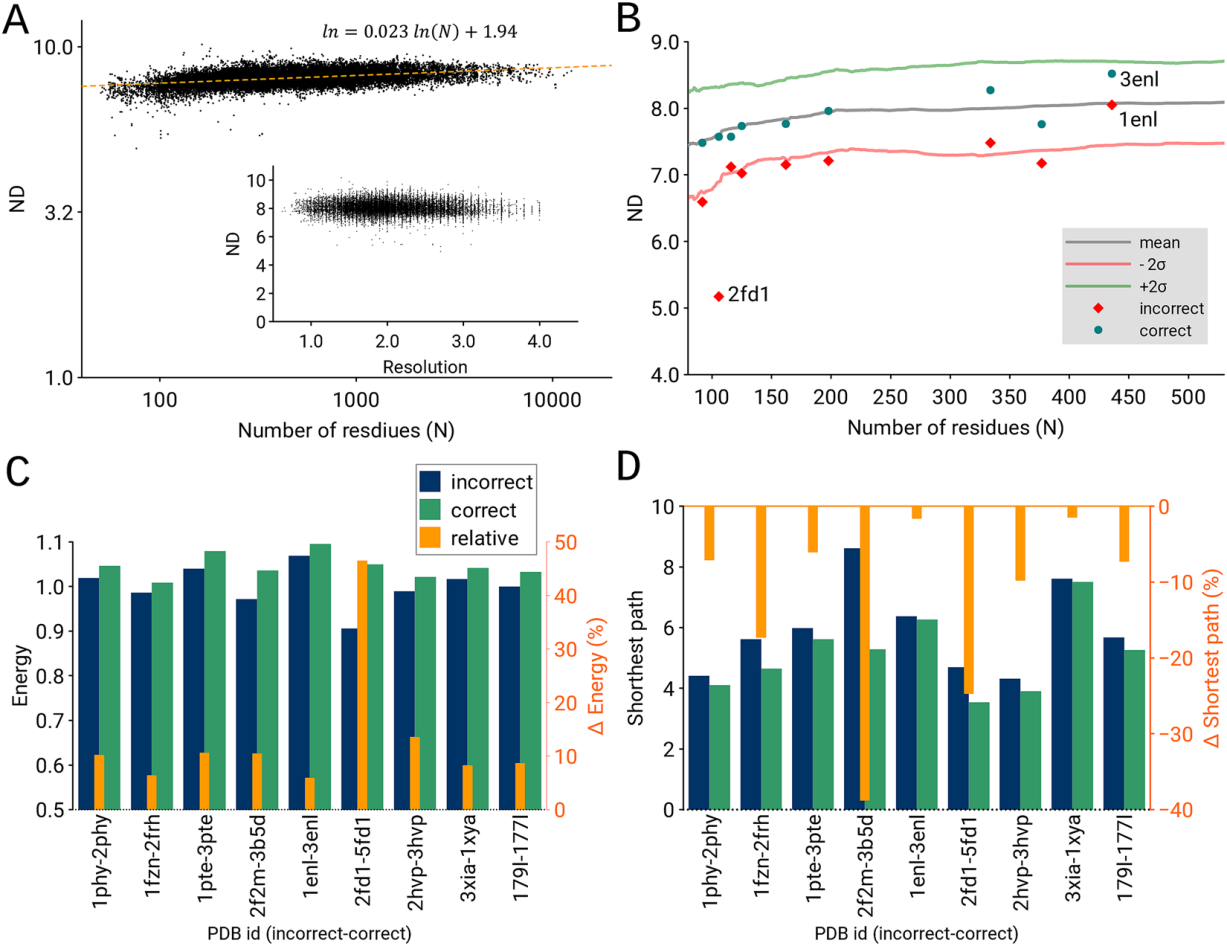
(**A**) Scaling exponent of node degree (ND) *versus* number of residues (N). (Inset) Distribution of ND against resolution data (Å) of the protein structures. (**B**) Mean ND, shown in grey, and ±2σ levels that approximately correspond to the 2.5 and 97.5 percentile levels, shown in green and red, respectively, are plotted smoothly, along with all individual data points for incorrect (red diamonds) and correct (blue circles) protein models. (**C**) Normalized graph energy and (**D**) shortest path length of nine pairs incorrect and correct structural models. The change in graph energy and shortest path length is shown in blue and green, respectively, whereas the relative change between the incorrect and correct model is shown in orange.

We analysed more than 50,000 residue networks and evaluated the dependence of node degree (ND) on (i) resolution and (ii) residue network size. The ND parameter indicates how an average node is connected to the other nodes. The distribution of ND clearly shows that ND does not depend on resolution (Fig. 1A, inset) and suggests that ND is not strongly related to protein size (N), as $ND\,{N}^{0.023}$ (Fig. 1A). Furthermore, the expected ND can be given by the expression $ND=7{N}^{0.023}$, where N is number of nodes, and the next approximation is given as ${e}^{1.94}\approx 7$. The resulting scaling exponent of 0.023 indicates that an increase in the protein size of 1000 times increases the ND by approximately 20% on average. Therefore, the ND of proteins of similar sizes are not randomly scattered over a wide range but rather are distributed in a narrow interval (σ ≈ 0.4). Note that a higher ND for large proteins may be observed because larger proteins have more core nodes. Moreover, larger proteins are made from several domains, thereby forming quaternary structures and hence establishing additional edges between adjacent domains in three-dimensional space^19^.

### Global correctness of macromolecular structures

**Figure 2:**
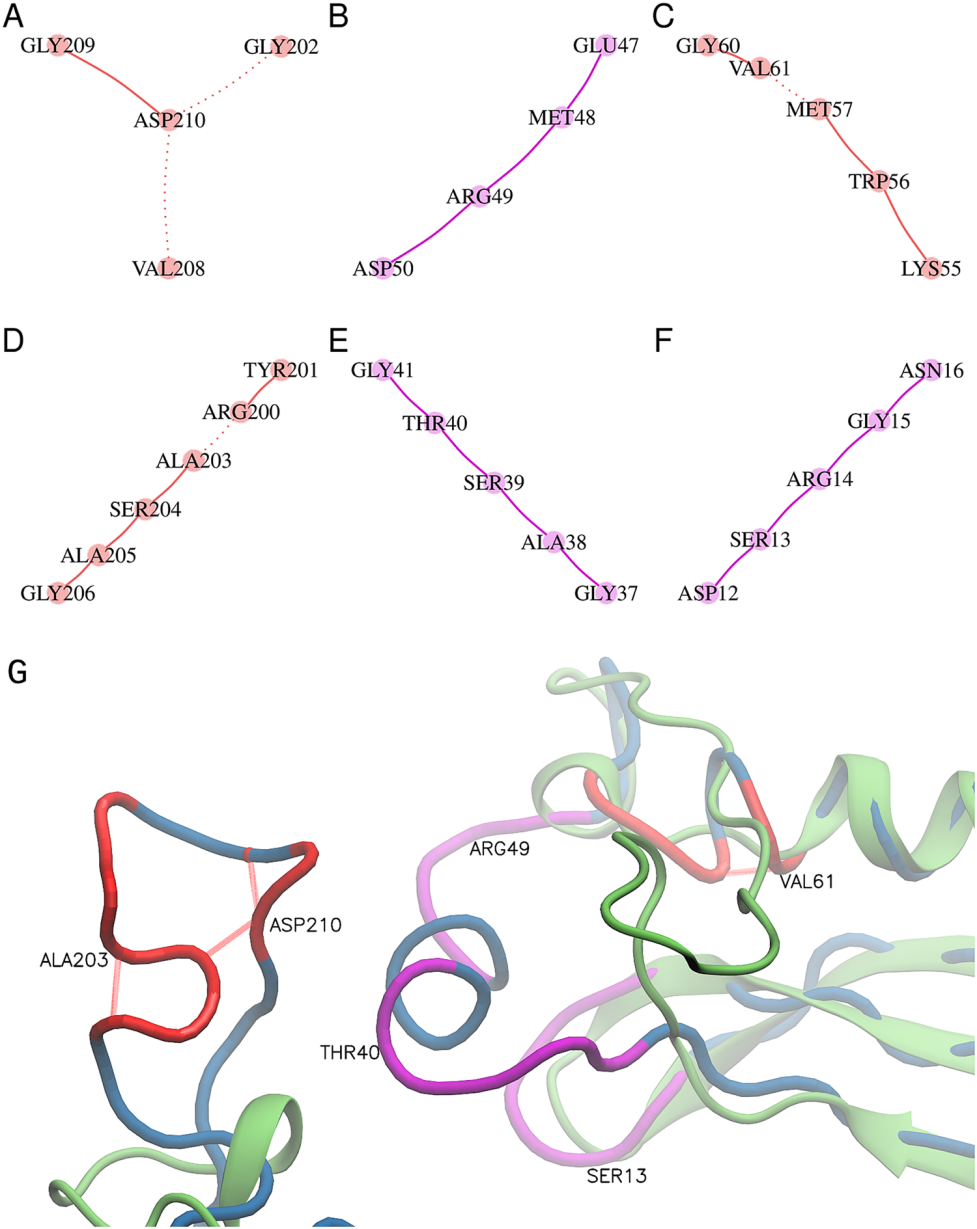
(**A**–**F**) Poor and long Cα subgraphs of an incorrect protein model (PDB id: 1ENL). Graphs coloured in red correspond to poor Cα subgraphs, while graphs coloured in magenta correspond to long Cα subgraphs. (**G**) Ribbon presentation of the correct structure in green (PDB id: 3ENL), and wire presentation of incorrect model in blue (PDB id: 1ENL). Colours and residue labels in the bottom panel correspond to the red and magenta subgraph colours. Poor edges (red) between non-sequential residues are plotted using dotted lines.

Because ND does not depend on resolution and its relation to protein size is rather weak, we investigated the general applicability of complex network parameters for validation and quality assessment. To address the global correctness of the model, we analysed the shortest path length and graph energy of nine pairs of previously studied correct and incorrect protein models (Supplementary Table 1). In graph theory, the degree of a node corresponds to the number of edges (i.e., the pair-wise contacts between residues), the shortest path length indicates how fast information spreads from a given node to other reachable nodes in the network, and the graph energy measures the stability of the connections in the network. The correct protein models have a significantly higher node degree (Fig. 1B), higher graph energy (Fig. 1C), and lower shortest path length (Fig. 1D). The relative change in graph energy ranges from 6% to 48%; the same is true for the shortest path length where a negative change is observed. Furthermore, the incorrect structures have node degrees approximately 2σ below the mean (Fig. 1B). The only exception among the presented cases is the pair (PDB id) 1ENL and 3ENL. The node degree of the incorrect model (PDB id: 1ENL) indicates that this model is in fact correct; however, the correct structure (PDB id: 3ENL) has an even higher node degree that is closer to the good percentile. Additional analysis of the poor and long Cα subgraphs of the incorrect model revealed a region of Cα atoms that are too close or too far apart from the expected distance (∼3.8 Å) (Fig. 2A–F). Note that in the case of poor subgraph construction, an edge is formed when the distance between any two Cα atoms is in the range of 3.0 to 3.7 Å. Inspection of the subgraphs of the incorrect structure (PDB id: 1ENL) revealed that the poor subgraphs contain edges between non-sequential amino acid pairs: Val208-Asp210, Gly202-Asp210, Met57-Val61 and Arg200-Ala203 (Fig. 2A,C,D). Furthermore, the ribbon representation (Fig. 2G) of the three poor and three long subgraphs of the incorrect model indicates a mistraced region. Thus, we have demonstrated that the poor and long Cα subgraphs (i.e., connected components) can identify problematic regions in the protein structure and can assist in the final part of model building.

**Figure 3:**
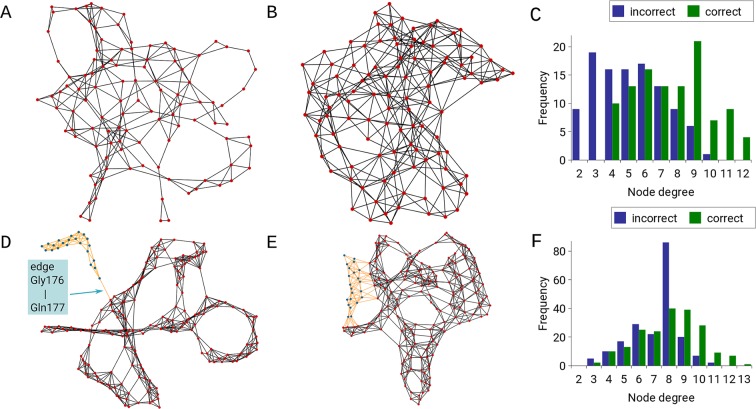
The 2D representations of two incorrect models (**A**,**D**) against their correct counterparts (**B**,**E**) using an organic layout visualized with Cytoscape (www.cytoscape.org). (**A**) Incorrect model (PDB id: 2FD1) and (**B**) correct counterpart (PDB id: 5FD1). (**D**) Incorrect model (PDB id: 2F2M) and (**E**) correct counterpart (PDB id: 3B5D), with the region around the Gly176-Gln177 edge shown in orange and marked. (**C**) Frequency of nodes against node degree of an incorrect (PDB id: 2FD1) and a correct protein model (PDB id: 5FD1) shown with blue and green bars, respectively. (**F**) Frequency of nodes against node degree of an incorrect (PDB id: 2F2M) and a correct protein model (PDB id: 3B5D) shown with blue and green bars, respectively.

Next, we analysed the case (PDB ids: 2FD1 and 5FD1) that shows the largest increase of node degree (Fig. 1B) and graph energy (Fig. 1C) among our examples. Visual inspection of the residue networks (Fig. 3A,B) shows that the correct model has a well-balanced distribution of edges that link the amino acids/nodes. The node degree distribution clearly shows that the correct structure has more nodes with a higher degree and does not have nodes with a very low degree (Fig. 3C). Thus, the correct model does not contain any nodes with a low degree but has 13 nodes with degree greater than 10. In contrast, the incorrect model contains 28 nodes (9 + 19) that have fewer than four edges and does not contain any nodes with degree greater than 10. In addition to the large increase in node degree and energy, the case (PDB ids: 2F2M and 3B5D), exhibits a pronounced decrease in the shortest path length (Fig. 1D). Visual inspection of the incorrect model revealed that a group of 22 residues (vertices in blue and edges in orange, Fig. 3D) are connected to the rest of the protein residues by only one link between two sequential residues, Gly176 and Gln177 (Fig. 3D). In the correct structure, the same group of 22 amino acids/nodes forms a considerably higher number of links (Fig. 3E); thus, the edges represent non-covalent interactions with other nodes in the residue network. Additionally, the node degree distribution shows that the incorrect model has a high peak at node degree 8 and only two nodes that have more than 10 edges (Fig. 3F). The correct model contains 17 nodes with a degree greater than 10 and with data evenly distributed about the mean^19^ (Fig. 3F).

### Identification of local errors

**Figure 4:**
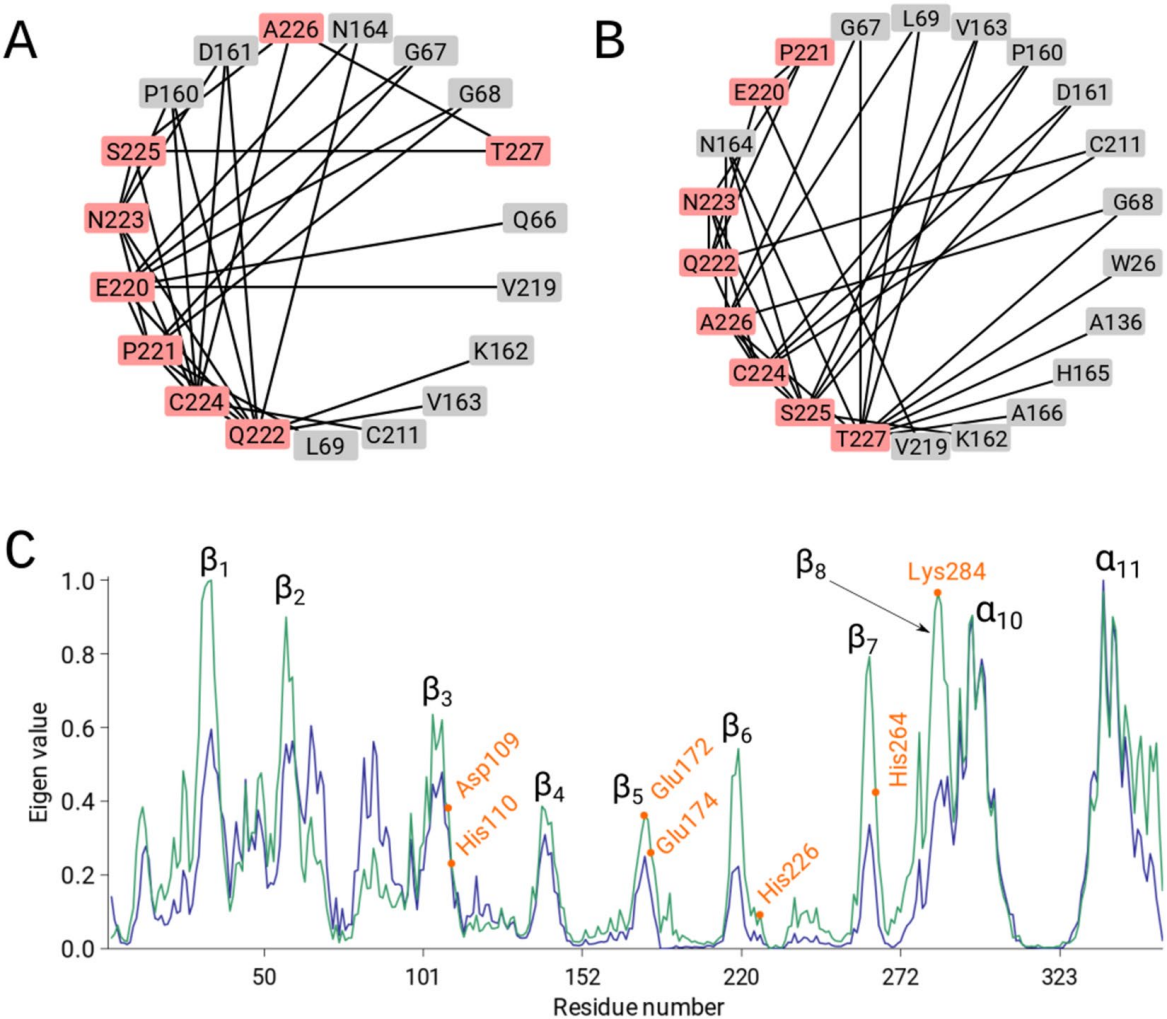
(**A**,**B**) The 2D representations of the residue network using a circular layout visualized with Cytoscape (www.cytoscape.org). The mini-chain nodes are coloured in red, whereas the body residues of Cathepsin H (PDB id: 8PCH) are coloured in grey. Edges are marked as black lines. (**C**) Normalized vector components (maximum score is set to 1) that correspond to the largest eigenvalue of the incorrect and correct structure, plotted against the residue index (PDB id: 1ZEN). The incorrect and correct structures are plotted in blue and green, respectively. The bimetallic-binding site residues in the (α/β)_8_ barrel are marked in orange.
(ii). The 1ZEN structure is partially incorrect due to several sequence register errors. Again, the global parameters show that the correct model has more edges and a lower shortest path length (Supplementary Table 2). In addition, we performed graph spectral analysis of the adjacency matrix for PDB id: 1ZEN. The eigenvector of the largest positive eigenvalue, i.e., eigenvector centrality, is a common measure of the importance of the nodes in the network. On the one hand, the eigenvector of the incorrect model exhibited very high scores in region of the α_10_ and α_11_ helices but had scores below 0.6 for rest of the structure (Fig. 4C). On the other hand, the eigenvector of the correct model exhibited the highest scores for β strands 1, 2, 7 and 8 (Fig. 4C), while also displaying high scores (>0.8) for the α_10_ and α_11_ helices. This analysis reveals that the eigenvector centrality of the correct structure provides a better interpretation and reveals the importance of the (α/β)_8_ barrel and the α_10_ and α_11_ helices in dimer formation (Fig. 5A–D). Additionally, the bimetallic binding site in the (α/β)_8_ barrel contains residues Asp109, His110, Glu172, Glu174, His226, His264 and Lys284, which have higher eigenvector values compared to the eigenvector values of these residues in the incorrect model (Fig. 4C).

Next, we inspected three cases with local problems: (i) an eight-residue-long bound peptide that can be traced in two alternative directions, (ii) a register error, and (iii) differences between non-crystallographic symmetry-related molecules.

(i). Protein cathepsin H (PDB id: 8PCH) has an eight-residue-long propeptide, termed a mini-chain, with a disulphide bond link to the main-chain. There are two alternative chain traces of the mini-chain. Using kick maps, the authors decided on the correct chain trace^26^. The ND analysis showed that the correct trace of the mini-chain is linked by 14 body residues, whereas the incorrect trace is linked by 11 body residues (Fig. 4A,B). Moreover, the high sensitivity of the global network parameters revealed a higher node degree and lower shortest path length for the model with the correctly placed mini-chain (Supplementary Table 2).

**Figure 5:**
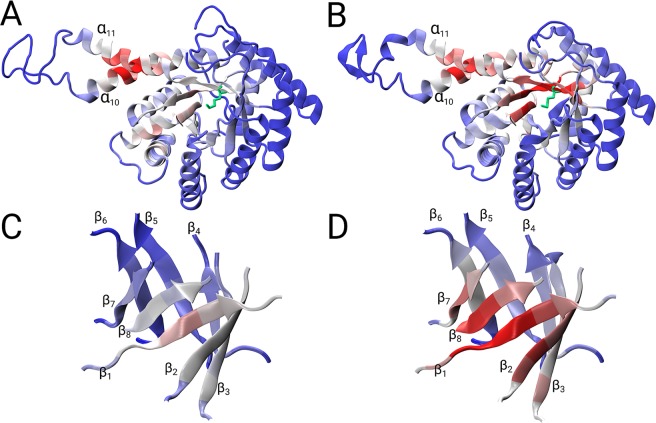
Ribbon presentations of vector components (maximum component is set to 1) that correspond to the largest eigenvalue of the incorrect (**A**,**C**) and correct structure (**B**,**D**). High vector values are coloured in red, whereas low vector values are coloured in blue; Lys284 is in green. (**C**,**D**) Show a magnified view of the (α/β)_8_ barrel.
(iii). Furthermore, we demonstrated the usefulness of the residue network parameter, e.g., node degree, to expose differences between two pairs of molecules in the asymmetric unit that are related by non-crystallographic symmetry (NCS). Molecules in a very similar environment are expected to have similar, but not identical, three-dimensional structures. The ND differences between two pairs of the same protein structure were calculated by subtracting the ND of chain A from the ND of chain B. The correct pair (PDB id: 1CEL) of structures exhibited insignificant discrepancies in ND along the chain (Fig. 6A), whereas the large ND discrepancies along the entire residue network in 3SDP (Fig. 6B) revealed that the previous lack of tools for the refinement of twinned crystals and the absence of NCS restraints hampered refinement in such cases. However, when the differences are localized, for example in allosteric enzymes, then the graph can indicate conformational differences between the relaxed and tense states. For example, analysis of the crystal structure of bacterial *L-lactate dehydrogenase*^27^ (PDB id: 1LTH) revealed that the region with the largest discrepancies involves residues Ala17, Pro89, Pro126, Ile229 and Ile230 (Fig. 6C). This insight is consistent with the findings of visual inspection, which revealed sites with large conformational changes.

**Figure 6:**
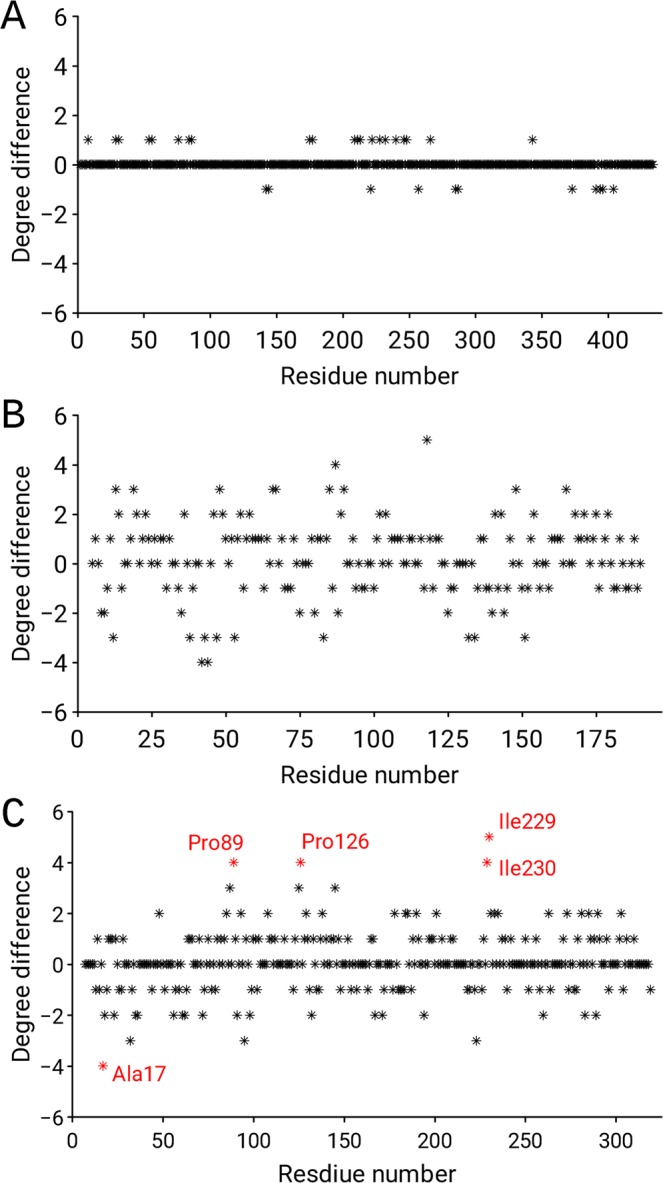
(**A**,**B**) Node degree differences between different copies of the same protein related by non-crystallographic symmetry. The plot reveals the distribution of (**A**) the correct model (PDB id: 1CEL), and (**B**) the incorrect model (PDB id: 3SDP). (**C**) Node degree differences between the relaxed and tense states of the crystal structure of bacterial L-lactate dehydrogenase (PDB id: 1LTH).

### Ranking decoys

In addition to the nine models that had been deposited in the PDB but later found to be incorrect we ranked the CASP11-stage1^28^ and Sali Lab^29^ decoy datasets. A total of 110 targets and their corresponding 7,800 models were taken in to our analysis. Energy statistical functions^30–34^ and Machine learning technique^35–39^ are often used on the decoy discrimination problem.

**Figure 7:**
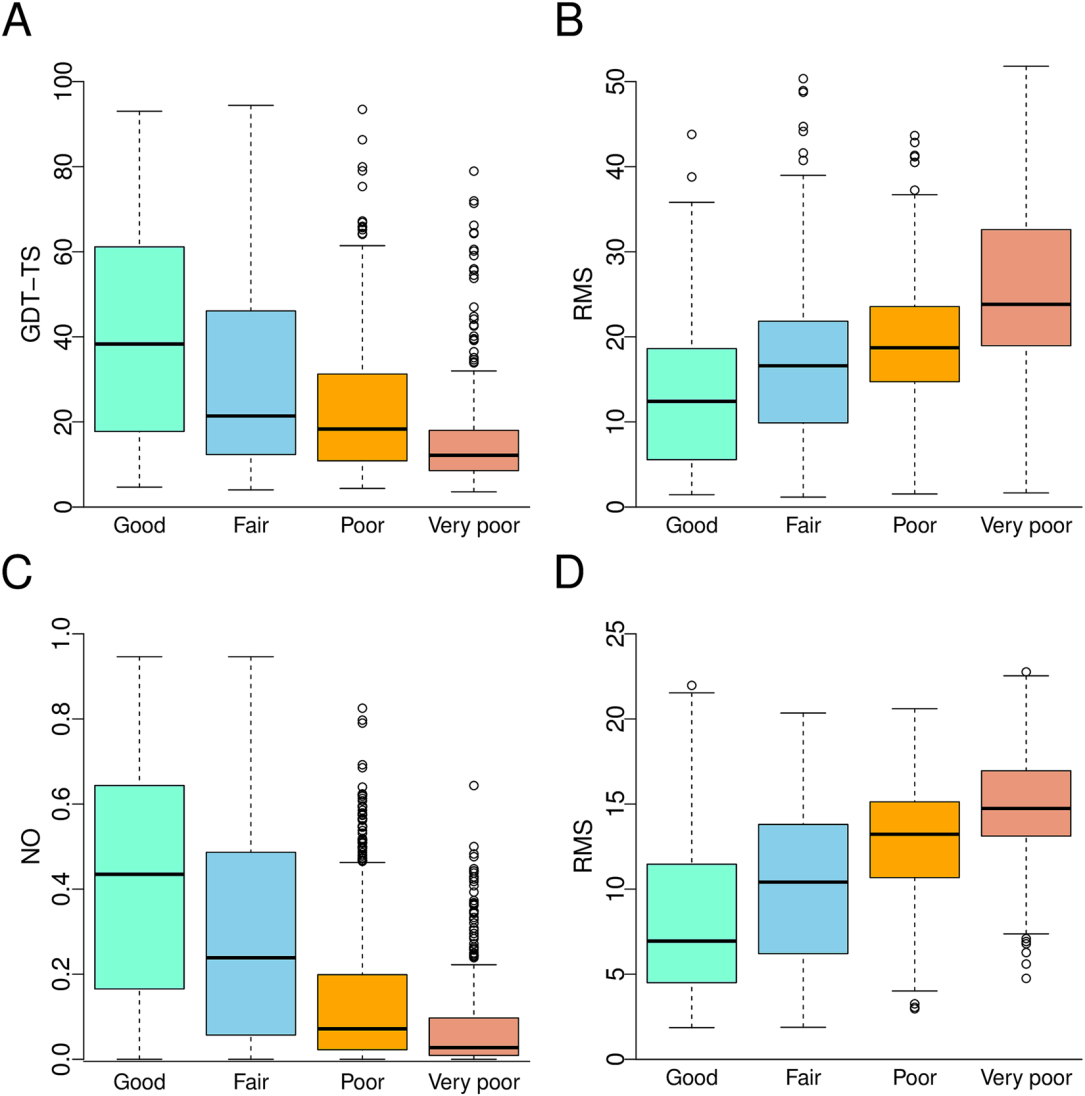
Boxplot of the GDT-TS, NO and RMSD for models in each quality group: Good - absolute value of the Z-score in the interval [0, 1], Fair - absolute value of the Z-score in the interval [1, 2], Poor - absolute value of the Z-score in the interval [2, 3], Very poor - absolute value of Z-score is greater than 3. (**A**,**B**) CASP11-stage1 and (**C**,**D**) Sali Lab dataset.

Here we introduce simple, but efficient ranking which is rather similar to the comparison of important global quality indicators in the wwPDB validation report. By using the Z-score of mean node degree we classified decoys in to four quality groups: (i) Good: absolute value of the Z-score in the interval [0, 1], (ii) Fair: absolute value of the Z-score in the interval [1, 2], (iii) Poor: absolute value of the Z-score in the interval [2, 3], (iv) Very poor: absolute value of Z-score is greater than 3. The Z-score of mean node degree was compared against three structural similarity metrics: global distance total score (GDT-TS)^40^, Root Mean Square Distance (RMSD) of Cα atoms and Native Overlap (NO)^41^ at a 3.5 Å cutoff. Note, the similarity metrics GDT-TS, RMSD and NO are part of the CASP11-stage1 and Sali Lab decoy datasets. Figure 7 shows the relation of classified decoys and the similarity metrics for the CASP11-stage1 (Fig. 7A,B) and Sali Lab (Fig. 7C,D) datasets. It can be seen that when the model has a very low (<−3) or a very high (>3) Z-score then the quality of the structure is low. Low quality structures have the highest RMSD and the lowest GDT-TS and NO. Models that have been assigned to the Good quality group show the lowest RMSD (Fig. 7B,D) and highest GDT-TS and NO (Fig. 7A,B). There is a positive (negative) trend for RMSD (GDT-TS, NO) when going from good towards very poor quality group. From the above examples, it is clear that by employing different parameters of the residue network, we were able to detect suspicious 3D models deposited in the PDB and to appropriately rank more than 7,800 decoys from the CASP11-stage1 and Sali Lab datasets.

Moreover, the evaluation of the presented method with standard metrics (average per-target correlation), shows that ND Z-score and shortest path strongly correlate with RMS (Supplementary Table 3). The comparison with DeepQA^38^, which is a single model quality assessment method, on the CASP11-stage1 dataset shows that DeepQA correlates (R = 0.64) better with GDT-TS, than residue graph parameters ND Z-score (R = 0.41) and shortest path (R = −0.39), see Supplementary Table 3. However, ND Z-score and shortest path correlate more closely than FUSION (R = 0.10) and raghavagps-gaspro (R = 0.35) on the CASP11-stage1 data set^38^. Furthermore, the correlations of DeepQA training feature spanned from 0.37 (Euclidean compact score) up to 0.63 (Qprobe score). It follows that ND and shortest path (absolute value) correlate better than the lowest DeepQA feature^38^ on the CASP11-stage1. In this respect, the results show that residue graph parameters could be used for ranking and selecting protein models as a single quality assessment method or as an individual feature of a deep belief network.

## Discussion

**Figure 8:**
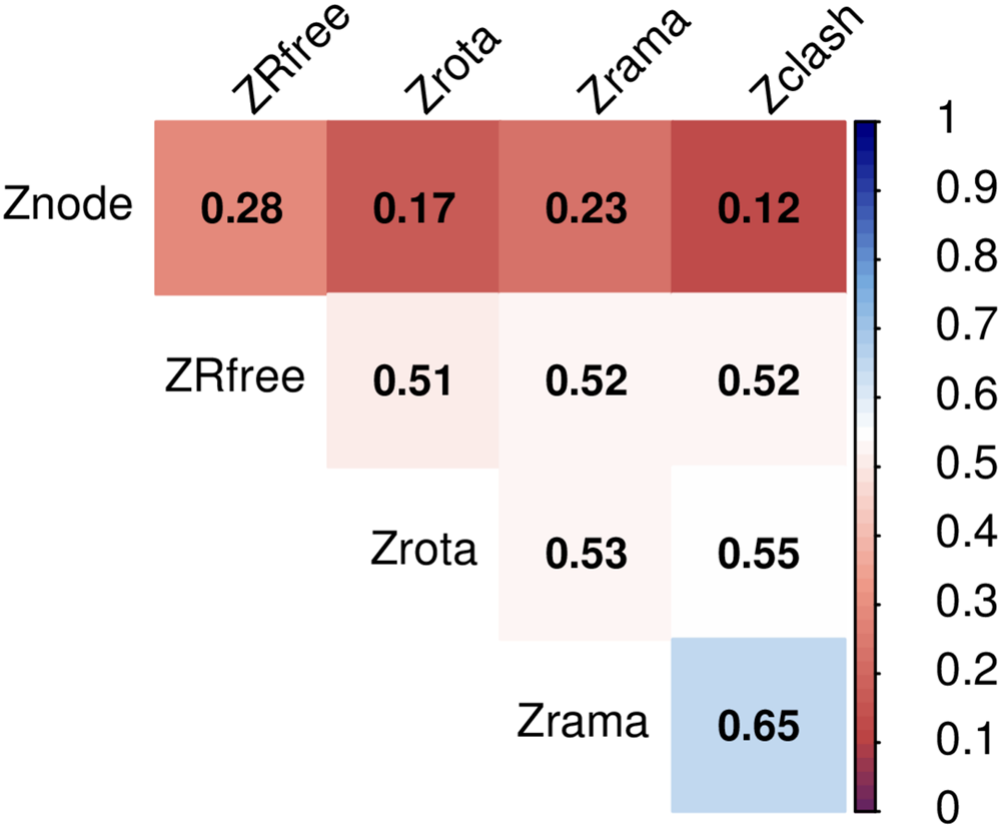
The correlation matrix shows correlations between following Z-scores: Ramachandran (Zrama), rotamer (Zrota), clashscore (Zclash), Rfree (ZRfee) and node degree (Znode). Ramachandran, rotamer, clashscore and Rfree Z-score was calculated from global percentile ranks taken from wwPDB X-ray Structure Validation Report. Node degree Z-score was calculated with respect to entries of similar length.

These examples demonstrate that the complex network analysis of a residue-based network is a useful tool for regional and global validation of three-dimensional macromolecular models. The standard validation criteria included in the wwPDB validation reports, i.e., Ramachandran, clash and rotamer Z-score exhibit moderate to strong correlation (R > 0.5, Fig. 8), meanwhile the comparison of the ND Z-score with Ramachandran, clash and rotamer Z-score, showed much lower correlation (R ~ 0.2). Our comparisons and the resolution independence of the network parameter analysis confirmed its independence from the usual local validation criteria, and the global criterion, such as the crystallographic R_free_ factor^3^. Importantly, the construction of a residue network from a macromolecular model is not included in the stereo-chemical restraints applied to the model during model building and refinement. Hence, the nonbiased approach is another key advantage of residue network analysis. The network analysis is also applicable for Cα-only models, which lack validation tools. The network analysis presented here is equally applicable to three-dimensional structures of macromolecules determined by electron microscopy. To conclude, we expect that the use and further development of the network analysis presented here will enhance the validation and quality assessment of three-dimensional structures, thereby deepening our understanding and insight into the biological functions of proteins.

## Methods

### Residue network database

Node degree and clustering coefficient against protein size and resolution was analysed using 50,249 residue networks. Residue network data were retrieved from the Protein Graph Repository^42^ (http://wjdi.bioinfo.uqam.ca/). By transformation of the three-dimensional protein models into the 2D graphs, each amino acid is abstracted as a Cα atom. The Cα atom of an amino acid residue is a node, and an edge is created when the distance between Cα atoms is equal to or below a threshold value of 7 Å^43^.

In an incorrect model graph, edges between nodes are constructed when the distance between any two nodes (Cα atoms) is between 3.0 and 3.7 Å. The edges in a long graph are constructed only when sequential nodes in the primary structure of protein model were more than 3.9 Å apart from each other. The threshold distances and poor/long terminology followed terminology introduced by Kleywegt, 1997^44^. Poor and long graphs were then decomposed into connected components, i.e., subgraphs.

The structures from the Protein Graph Repository were selected according to the following criteria: (i) the protein chain was longer than 50 residues, (ii) the resolution of the crystallographic experimental data was beyond 4.0 Å and (iii) the protein was a member of the Structural Classification of Proteins (SCOP)^45^ classes A (α proteins), B (β proteins), C (α/β proteins), D (α + β proteins), E (multi-domain proteins) or F (membrane and cell surface proteins and peptides), see Supplementary Material for details on the raw data sets.

### Incorrect and correct protein models

An objective comparison between two residue networks can only be made when they have the same number of nodes, which excludes comparisons of models with different numbers of missing residues. Before performing a detailed analysis of the network parameters, we constructed Cα atom matches between the incorrect and correct models. The incorrect and correct primary structures were aligned, and only the residues present in both models were used to construct the residue network.

All network parameters were calculated using online tool for Network based Analysis of Protein Structures^46^, Bio3D (2.3.3) and igraph (1.0.1) R package (3.4.0), see Supplementary Material for details on R scripts.

Protein models (PDB ids) 1PHY, 1PTE, 1ENL, 2FD1, 3XIA and 179l are obsolete in the Protein Data Bank (PDB) and have been superseded by 2PHY, 3PTE, 3ENL, 5FD1, 1XYA, and 177l, respectively. The structure of aspartyl protease from human immunodeficiency virus HIV-1 (PDB id: 2HVP) is partly incorrect and was later corrected and refined as chemically synthesized HIV-1 protease (PDB id: 3HVP). Structures (PDB id) 1FZN and 2F2M are both obsolete but have not been superseded. In *Crystal structures of SarA*, *a pleiotropic regulator of virulence genes in S*. *aureus*^47^ (PDB id: 1FZN), an erratum was published. Later, in 2006, the correct model (PDB id: 2RH) was deposited into the PDB database^48^. The X-ray structures of EmrE (PDB id: 2F2M and 3B5D) was published by Chang and co-workers. The structure published in 2006 (PDB id: 2F2M) was later retracted, and the correct model was published in 2007^49^ (PDB id: 3B5D).

The three additional cases were selected from our previous works in which all three protein models were analysed. The structure of Cathepsin H with both the correctly and reversely built mini-chain (PDB id: 8PCH) was analysed by validation of averaged kick maps, which can remove model bias from a protein model^26,50^. The crystal structure of a class II fructose-1,6-bisphosphate aldolase (PDB id: 1ZEN) is partly incorrect and contains a registry error. This case was analysed during method validation for the removal of model bias^50–52^. Furthermore, this partly incorrect model was rebuilt, refined and used to show the efficacy of free kick refinement^52^. The comparison and presentation of the correct model (PDB id: 1CEL) and the model with unusual features (PDB id: 3SDP) related by non-crystallographic symmetry was a case in *Validation of protein crystal structure*^1^.

### Graph

A graph *G* = *G(V*, *E)* consists of a set of vertices (nodes) *V* = *v*_1_, *v*_2_, *… v*_*n*_ and a set of edges *E* = *e*_1_, *e*_2_, *… e*_*m*_. Two vertices *v*_*i*_ and *v*_*j*_ of a graph *G* are said to be adjacent if there is an edge *e*_*ij*_ connecting them. The vertices *v*_*i*_ and *v*_*j*_ are then said to be incident to the edge *e*_*ij*_. Two distinct edges of *G* are adjacent if they have at least one vertex in common.

### (Average) node degree

The degree of a node *v*, denoted *d(v*), represents the number of nodes adjacent to *v*. The average node degree of a graph *G* is the average value of the degrees of all nodes in *G*. The average node degree is formally written as

$$d(G)=\frac{1}{N}\sum _{i=1}^{N}d({v}_{i})$$

where *d(v*_*i*_*)* represents the degree of the node *v*_*i*_ and *N* is the total number of nodes in a graph *G*. Another way of expressing the average node degree is with the ratio

$$d(G)=\frac{2e(G)}{N(G)}$$

where *e(G)* represents the total number of edges in a graph *G*, and *N(G)* is the number of nodes in a graph *G*.

### Average shortest path

Let *G* = *G(V*, *E)* be a graph containing *n* vertices and *m* edges, with the set of vertices V = *v*_1_, *v*_2_, *… v*_*n*_ and the set of edges *E* = *e*_1_, *e*_2_, *… e*_*m*_. The adjacency matrix *A(G*) = (*a*_*i*,*j*_) of *G* is then a *n* × *n* matrix defined by

$${a}_{i,j}=\{\begin{array}{c}1,if({v}_{i},\,{v}_{j})\in E\\ 0,\,otherwise\end{array}$$

Since protein graphs are simple graphs without loops or multiple edges, the adjacency matrix of any protein graph is symmetric with zeros on the diagonal. The shortest path between two nodes, *v*_*i*_ and *v*_*j*,_ is the minimal number of edges that lie between two given nodes. For computing the shortest path between a pair of nodes, we used Dijkstra’s algorithm^53^.

### Largest eigenvalue (LEV) and corresponding eigenvector (eigenvector centrality)

The adjacency matrix *A(G)* of an undirected simple graph is symmetric and therefore has a complete set of real eigenvalues and an orthogonal eigenvector basis. The set of eigenvalues of a graph is the spectrum of the graph. Eigenvalues are denoted as *λ*_1_ ≥ *λ*_2_ ≥ *… λ*_*N*_. The eigenvalues are obtained as the roots of the characteristic polynomial of matrix *A*; that is, we look for the solutions of the equation

$$det(A-\lambda I)=0$$

where *I* is the identity matrix. For every eigenvalue, we can identify at least one vector ***x*** for which it holds

$$\lambda x=Ax$$

where vector ***x*** is called a corresponding eigenvector of the given eigenvalue *λ*, and *A* is an adjacency matrix. The Perron–Frobenius theorem asserts that a real square matrix with positive entries has a unique largest real eigenvalue and that the corresponding eigenvector can be chosen to have strictly positive components. The *v*^*th*^ component of the vector that corresponds to the largest eigenvalue (*λ*_*1*_ = *LEV*) gives the relative centrality score of the vertex *v* in the network.

### Energy of a graph

The graph energy *E(G)* of a graph *G* on *N* vertices with *M* edges is defined as the sum of the absolute values of all eigenvalues *λ*_*1*,_ *λ*_2_ *…*, *λ*_*N*_ of the adjacency matrix *A(G)*, formally written as

$$E(G)=\sum _{i=1}^{N}|{\lambda }_{i}|$$

## Supplementary information

Supplementary material

Dataset 1
