# Supplementary material for: Validation and quality assessment of macromolecular structures using complex network analysis
Source: Sci Rep. 2019 Feb 8;9:1678. doi: 10.1038/s41598-019-38658-9 (PMC6368557; doi:10.1038/s41598-019-38658-9)
Supplement: Supplementary file 1 — Supplementary material [file 41598_2019_38658_MOESM1_ESM.docx]

**Supplementary Material**

**Validation and quality assessment of macromolecular structures using complex network analysis**

Jure Pražnikar^1,2*^, Miloš Tomić^1^, Dušan Turk^2,3^

^1^Faculty of Mathematics, Natural Sciences and Information Technologies, University of Primorska, Slovenia.

^2^Institute Jožef Stefan, Slovenia.

^3^Center of excellence for Integrated Approaches in Chemistry and Biology of Proteins, Slovenia.

**Supplementary Table S1.** PDB ids of the nine protein model pairs. Each pair has a poor and a good protein model.

| **Poor** | | | | | | | | |  |
| --- | --- | --- | --- | --- | --- | --- | --- | --- | --- |
| **PDB id** | 1PHY | 1FZN | 1PTE | 2F2M | 1ENL | 2FD1 | 2HVP | 3XIA | 179L |
| **Residues**  **per chain** | A: 126 | A: 123 | A: 348 | A: 113  B: 113 | A: 436 | A: 106 | A: 99 | A: 388 | A: 162 |
| **Good** | | | | | | | | |  |
| **PDB id** | 2PHY | 2FRH | 3PTE | 3B5D | 3ENL | 5FD1 | 3HVP | 1XYA | 177L |
| **Residues**  **per chain** | A: 125 | A: 127  B: 127 | A: 349 | A: 110  B: 110 | A: 436 | A: 106 | A: 99 | A: 386  B: 386 | A:162 |
| **Cα match** | | | | | | | | |  |
| **Nodes** | 125 | 116 | 334 | 198 | 436 | 106 | 92 | 377 | 162 |

**Supplementary Table S2.** Number of nodes, number of edges and shortest path value for poor and good protein models.

| PDB id | Nodes | Edges | Shortest path |
| --- | --- | --- | --- |
| *poor –* **good** | | | |
| 8PCH | *228 –* **228** | *921 –* **925** | *4.60* – **4.58** |
| 1ZEN | *338 –* **338** | *1370 –* **1404** | *6.19* – **6.06** |

**Supplementary Table S3.** Average per target correlation between ND Z-score (shortest path) and GDT-TS (RMS) on CASP11-stage1 dataset. Additionally, DeepQA and DeepQA training feature correlations on CASP11-stage1 dataset are shown. *Data from DeepQA: improving the estimation of single protein model quality with deep belief networks. BMC Bioinformatics 17, 2016.

|  | ND Z-score | Shortest Path | DeepQA | DeepQA training feature |
| --- | --- | --- | --- | --- |
| RMS | -0.62 | 0.74 | / | / |
| GDT-TS | 0.41 | -0.39 | 0.64* | [0.37-0.63]* |

**Selected protein graphs from the repository**

The protein graphs from the Protein Graph Repository (<http://wjdi.bioinfo.uqam.ca/>) were selected according to the following criteria:

1. the protein chain is longer than 50 residues
2. the resolution of the crystallographic experimental data is beyond 4.0 Å and
3. the protein is a member of the Structural Classification of Proteins classes A, B, C, D, E or F

(File name: raw_PGR_data.csv)

**Smooth data**

Smooth mean and standard deviation was calculated using data source (raw_PGR_data.csv), with a window length [0.8N, 1.2N], where N is the length of protein (File name: SmoothData.csv).

**Z-score calculation**

Ramachandran, rotamer and clash Z-score was calculated using R package whit following one-line script:

z <- qnorm(p)

where z is Z-score and p is percentile rank taken form wwPDB validation report.

**Mean node degree, average shortest path and graph energy**

Next R script was used to calculate mean node degree, average shortest path and normalized graph energy:

ash <- average.path.length(g) # average shortest path of graph g

mnd <- mean(degree(g)) # calculate mean node degree of graph g

myeigen <- eigen(adj) # input is adj matrix, calculate eigen values and vectors

EV <- (myeigen$values) # extract eigen values

energy <- sum(abs(EV)) # Graph energy is defined as sum of absolute eigen values

Nenergy <- energy/vcount(g)/2 # normalized graph energy

**Protein model analysis**

The R script (File name: ProtModAna.r) was used to analyse protein models. The script reads PDB file, converts 3D protein model into graph and calculates mean node degree, Z-score, poor and long subgraphs.
